# Probiotics and in-hive fermentation as a source of beneficial microbes to support the gut microbial health of honey bees

**DOI:** 10.1093/jisesa/iead093

**Published:** 2023-12-06

**Authors:** María A Rodríguez, Leticia A Fernández, Brendan A Daisley, Francisco J Reynaldi, Emma Allen-Vercoe, Graham J Thompson

**Affiliations:** Laboratorio de Estudios Apícolas (LabEA-CIC), Departamento de Agronomía, Universidad Nacional del Sur (UNS), Bahía Blanca, Buenos Aires, Argentina; Comisión de Investigaciones Científicas de la Provincia de Buenos Aires (CIC), Buenos Aires, Argentina; Department of Biology, The University of Western Ontario, London, ON, Canada; Laboratorio de Estudios Apícolas (LabEA-CIC), Departamento de Agronomía, Universidad Nacional del Sur (UNS), Bahía Blanca, Buenos Aires, Argentina; Departamento de Biología, Bioquímica y Farmacia, Universidad Nacional del Sur (UNS), Bahía Blanca, Buenos Aires, Argentina; Consejo Nacional de Investigaciones Científicas y Técnicas (CONICET), Buenos Aires, Argentina; Department of Biology, The University of Western Ontario, London, ON, Canada; Department of Molecular and Cellular Biology, University of Guelph, Guelph, ON, Canada; Consejo Nacional de Investigaciones Científicas y Técnicas (CONICET), Buenos Aires, Argentina; Centro de Microbiología Básica y Aplicada (CEMIBA), Facultad de Ciencias Veterinarias, Universidad Nacional de La Plata (UNLP), La Plata, Buenos Aires, Argentina; Department of Molecular and Cellular Biology, University of Guelph, Guelph, ON, Canada; Department of Biology, The University of Western Ontario, London, ON, Canada

**Keywords:** *Apis mellifera*, beneficial microbe, fermented food, water kefir, microbial ecology

## Abstract

Managed populations of honey bees (*Apis mellifera* Linnaeus; Hymenoptera: Apidae) are regularly exposed to infectious diseases. Good hive management including the occasional application of antibiotics can help mitigate infectious outbreaks, but new beekeeping tools and techniques that bolster immunity and help control disease transmission are welcome. In this review, we focus on the applications of beneficial microbes for disease management as well as to support hive health and sustainability within the apicultural industry. We draw attention to the latest advances in probiotic approaches as well as the integration of fermented foods (such as water kefir) with disease-fighting properties that might ultimately be delivered to hives as an alternative or partial antidote to antibiotics. There is substantial evidence from in vitro laboratory studies that suggest beneficial microbes could be an effective method for improving disease resistance in honey bees. However, colony level evidence is lacking and there is urgent need for further validation via controlled field trials experimentally designed to test defined microbial compositions against specific diseases of interest.

## Introduction

The European honey bee *Apis mellifera* is among the most commercially important insects to human well-being; it is heavily utilized in crop pollination services, an agricultural role that is essential to sustaining a fresh and healthy food supply (Klein et al. 2007). Despite this role, the global population of honey bees suffer from high overwinter mortality and it has become increasingly difficult to manage these insects in a sustainable manner (Potts et al. 2010, Vanbergen and Initiative 2013, Daisley et al. 2022a). There is therefore worldwide interest in developing new tools and techniques that, if practical, can complement best practice in commercial and small-scale beekeeping (Steinhauer et al. 2021).

One major factor that challenges bee vitality on a global scale is exposure to pests and pathogens, which require skilled monitoring and management to stave off loss of product or loss of whole colonies (McMenamin and Genersch 2015). The control of contagious disease through prescribed application of antibiotics is well known in beekeeping but this practice, though well intended, can adversely affect the bee’s own gut microbiome. The composition of the honey bee gut microbiome varies with caste, age, and environment (Martinson et al. 2012, Jones et al. 2018) but generally consists of a core set of relatively few (6–10) dominant phylotypes (Bonilla-Rosso and Engel 2018) that, when unperturbed, represent a steady state of bee gut symbioses (Moran et al. 2012). Microbial imbalance can thus disrupt the bee’s ability to activate innate defenses, extract nutrients from feed or to detoxify environmentally acquired pollutants (Daisley et al. 2020a, Chmiel et al. 2021). Moreover, the routine application of antibiotics can in the long term generate resistant pathogens (Roberts 1996, Piva et al. 2020, Obshta et al. 2023) and lead to further off-target effects in the hive, such as trace pharmaceutical residue in wax and honey. Ironically, application of antibiotics can indirectly increase susceptibility to other diseases (Raymann et al. 2017, Daisley et al. 2020a, Powell et al. 2021). For these reasons, access to antibiotics is increasingly restricted (Croppi et al. 2021).

Probiotics represent a relatively new approach to disease management that may circumvent some of these issues and otherwise improve the health of managed honey bee colonies (Vásquez et al. 2012, Alberoni et al. 2016, Alonso-Salces et al. 2017, Abdi et al. 2023), provided the science behind this effort is sound and fully published in scientific journals (Chmiel et al. 2021, Damico et al. 2023). For example, probiotics may bolster queen productivity, stimulate innate immune responses in larvae or adults, or otherwise provide functional support to the bee’s own native microbiome (Daisley et al. 2020a). These beneficial effects may be especially helpful to beekeepers following dysbiosis from antibiotic treatment or another disease-associated setback (Raymann et al. 2017, Daisley et al. 2020c). Here we highlight progress relevant to the control of microbial diseases in beekeeping, with a focus on American foulbrood and other gut-borne diseases that afflict honey bees. We draw attention to kefir and other fermented products as a potential source of beneficial microbes with disease-fighting properties that might ultimately be integrated into apicultural management strategies.

## Probiotic Approaches to Control Bee Disease

A myriad of disease-causing pests, parasites, and pathogens can impact honey bee survival (Hristov et al. 2020). From a microbial standpoint, the organisms primarily involved in colony loss include those of bacterial (e.g., *Paenibacillus larvae*, *Melissococcus plutonius*), fungal (e.g., *Ascosphaera apis*, *Vairimorpha* [*Nosema*] *ceranae*), and viral (e.g., deformed wing virus, acute bee paralysis virus, chronic bee paralysis virus) origin. Recent reviews have comprehensively characterized the global distribution (Pasho et al. 2021) and virulence mechanisms of these microbial pathogens (Boncristiani et al. 2021). So far, probiotic studies have mostly assessed the effect of oral supplementation (especially lactic acid bacteria) and focused on diseases that infect the honey bee intestinal tract such as American foulbrood (AFB), European foulbrood (EFB), chalkbrood, and Nosemosis.

### American Foulbrood

American foulbrood is a larval disease caused by the spore-forming bacterium *Paenibacillus larvae* (Genersch, 2010). The onset of disease occurs when spores, once ingested by young bee larvae (first or second instar), germinate in the midgut and proliferate in their vegetative growth phase. Bacteria then invade the hemocoel and degrade the larvae into a brown, glue-like liquid (De Graaf et al. 2006, Yue et al. 2008). If symptoms are not recognized and colonies incinerated, the pathogen can remain an active source of new infections and can spread to distant hives (Lindström et al. 2008). Further, as host nutrients are exhausted and diseased comb dries out, the pathogenic bacteria can encyst onto beekeeping equipment in the form of endospores that may lay dormant with the potential to re-activate for decades (Hasemann 1961). *Paenibacillus larvae* is therefore a significant pathogen of honey bee larvae (Ebeling et al. 2016).

What factors trigger the onset of asymptomatic-to-symptomatic foulbrood in living hives can vary with genotype (ERIC types I-IV) and is somewhat enigmatic (Alvarado et al. 2013) but may include interactions between *P. larvae* and other microbes found in the bee gut or broader hive environment (Erban et al. 2017, Daisley et al. 2022b), as appears to be the case for pathogen *Melissococcus plutonius* and the onset of a similar disease, European foulbrood (Floyd et al. 2020). Bee management would ideally consider the broader microbial ecology of host symbionts and invasive pathogenic strains within a larval or adult bee’s gut. This systems approach to bee management is consistent with an emerging theory in evolutionary biology, the holobiont theory, which considers hosts – in this case, bees – and their co-adapted microbes “as one” under selection (Guerrero et al. 2013). The systems level approach makes biological sense; bee guts are a stable environment full of nutrients that provide ample substrate for microbial symbioses (Gilliam 1997). Endogenous bee gut microbes play a role in digestion, detoxification, nutrient conversion, and resistance to pests and pathogens (Raymann and Moran 2018). If the health of bee and gut microbes are functionally codependent, then probiotic supplementation that supports and restores this function seems an effective way to improve bee health or otherwise mitigate the worst effects of disease (Vásquez et al. 2012, Alberoni et al. 2016, Alonso-Salces et al. 2017, Abdi et al. 2023, Ye et al. 2023).

Laboratory studies using lactic acid-producing bacteria have begun to test this hypothesis. One approach has been to use midgut extracts (e.g., Riessberger-Galle et al. 2001) or isolate single strains from the adult or larval honey bee’s native microbiome, then use in-lab screens to test for evidence of anti-pathogenicity against *P. larvae* (e.g., Evans and Armstrong 2005, Lee et al. 2009, Sabaté et al. 2009, Forsgren et al. 2010, Audisio et al. 2011, Killer et al. 2014, Arredondo et al. 2018, Bartel et al. 2019, Iorizzo et al. 2020b). The controlled study of individual strains helps to identify those that inhibit pathogen growth or that otherwise might increase survivorship of honey bee larvae that are reared and infected in vitro. Lab-based studies are necessarily removed from a natural hive and thus the “as one” aspect is suspended in favor of a deliberately separated approach. Culture assays on their own cannot assess any emergent gut microbiome community effects (e.g., McNally et al. 2014) nor any host-mediated effects (e.g., effects of immune system, microbiome dysbiosis, and nutrition; Daisley et al. 2022b) but are nonetheless efficient – at least 250 strains of mostly lactic acid bacteria have been screened for probiotic potential – and important first steps toward whole-colony field trails that, when warranted, will prompt the eventual development of bee-tailored probiotic products.

Other studies have screened strains from nonnative sources, such as isolating probiotic strains from fermented food (Yoshiyama et al. 2013) or from plants (Flesar et al. 2010, González and Marioli 2010), while others still combine strains from native and nonnative sources. Daisley et al. (2020b) showed using in vitro trials that a 3-strain lactic acid bacterial mixture (*Lactiplantibacillus plantarum* Lp39, *Lacticaseibacillus rhamnosus* GR-1, and *Apilactobacillus kunkeei* BR-1), which was dubbed “LX3”, could reduce pathogen load and improve larval survival following deliberate infection with *P. larvae*. Moreover, this and a subsequent study (Daisley et al. 2020c) found that in-hive supplementation with LX3 containing the native *L. kunkeii* decreased pathogen load in larvae and in adult carriers, and was equally or more effective at doing so than antibiotic (oxytetracycline) treatment.

### Other Gut-Borne Diseases

Chalkbrood is caused by a spore-forming fungus, *Ascosphaera apis*; an obligate specialist pathogen that infects honey bee larvae. The disease tends to afflict colonies that are already under nutritional or environmental stress and, while it does have serious consequences to beekeepers (Aronstein and Murray 2010), it does not usually kill whole colonies (Gilliam 1986). The development of chalkbrood disease is initiated when larvae ingest sexually produced spores, which then germinate in the lumen of the larval gut (Bamford and Heath 1989). Fungal hyphae then penetrate the intestinal walls and cover the larva with a layer of (usually) white—hence, “chalk”—mycelium (Aronstein and Murray 2010). New spores are formed on the cuticle of the cadavers, which can spread within and between colonies. Research into microbial intervention as a control for chalkbrood is not as advanced as for (the more virulent) bacterial infections (Wood 1998) but milder disease caused by fungal infections have nonetheless received focused attention (Vojvodic et al. 2012).

Screens for bacterial strains from adult worker guts that inhibit the chalkbrood pathogen have begun. Iorizzo et al. have shown a role for *Lactobacillus kunkeei* (Iorizzo et al. 2020a) and for *Lactiplantibacillus plantarum* (Iorizzo et al. 2021) to suppress *A. apis* growth in vitro. They tested multiple strains of each species, delivered in different types of preparations, to reveal that delivery of probiotic cells in pellet was especially efficacious against *A. apis* in culture. The authors suggest that these 2 strains (at least) hold promise as a microbial means to restore symbiotic communities of the intestine and exert a prophylactic action against chalkbrood infection. Meanwhile, these and other studies (Omar et al. 2014, Bartel et al. 2019, Iorizzo et al. 2020a) suggest that lactic acid bacteria hold potential as probiotics to control chalkbrood disease.

*Vairimorpha* (*Nosema*) *ceranae* is an intracellular spore-forming microsporidian parasite (Tokarev et al. 2020) of bees. Adult honey bees become infected by consuming *Vairimorpha* spp. spores that then germinate in the ventriculus to infect epithelial cells within the midgut. Infection causes lesions, suppresses humoral and cellular defenses, and leads to a decrease in vitellogenin expression (Fries 2010, Piano et al. 2017). Ptaszyńska et al. (2016) investigated the effect of *Lactobacillus rhamnosus* (a commercial probiotic) and inulin (a prebiotic) on the survival rates of honey bees infected and uninfected with *Nosema ceranae*. They report that honey bees feed sugar syrup supplemented with the pro- and prebiotic were more susceptible to *V. ceranae* infection. This unexpected result is however juxtaposed with findings from other studies. Borges et al. (2021) tested the effect of several commercial probiotics on *V. ceranae* spore loads and honey bee survivorship, finding that the probiotic treatments tended to decrease *V. ceranae* infections. Other studies have tested endogenous strains in-hive to show dampening effects on parasite load (Corby-Harris et al. 2016) and positive effects on other parameters relevant to beekeepers, including queen productivity and honey yield (Audisio et al. 2015, Audisio 2017, Arredondo et al. 2023).

## Fermentation in the Hive

Foods contaminated with pathogenic microorganisms are a common source of infection for many types of animals. In the human diet, deliberate fermentation with lactic acid-producing bacteria (e.g., *Lactobacillus*, *Lactococcus*, *Streptococcus*, *Bifidobacterium*, *Leuconostoc* spp.), can, however, prevent pathogenic infection, prolong food shelf-life, and confer other benefits to hosts that consume fermented foods (Table 1). Several approaches are possible, including lactic fermentation (e.g., yogurt, sauerkraut), yeast-lactic fermentation (e.g., kefir, sourdough bread), and mold-lactic fermentation (e.g., various cheeses), among others (Martins et al. 2013, Ranadheera et al. 2017, Aspri et al. 2020). For honey bees, lactic-yeast fermentation is naturally initiated in hives to produce bee bread from foraged pollen. As a result of fermentation, flavonoid content of pollen can increase (Kaškonienė et al. 2018), and bees seem to employ fructophilic lactic acid bacteria, such as *Apilactobacillus kunkeei*, *Fructobacillus fructosus*, *Lactobacillus plantarum*, and *Leuconostoc mesenteroides*, as evidenced by their detection in beebread (Ispirli and Dertli 2021). Further, the slow-growing yeast *Metschnikowia* spp. can breakdown sucrose and other disaccharides found in nectar, making it easier for bees to digest (Martin et al. 2022). The gram-negative bacteria *Gilliamella apicola* can also breakdown sugars and help to detoxify diet (Zheng et al. 2016), and natural fermentation chambers in hive can promote social transmission of beneficial microbes within the plant-pollinator network (Anderson et al. 2013).

**Table 1.** Properties of beneficial microorganisms isolated from diverse fermented products

Strain	Origin	Reported properties	Reference
*Lactobacillus plantarum, Lactobacillus fermentum, Lactobacillus delbrueckii*	Water kefir and braga (a Romanian fermented beverage made of cereals)	Antibacterial activity against pathogenic bacteria*: Listeria monocytogenes, Escherichia coli, Staphylococcus aureus,* and *Salmonella enterica*.	(Angelescu et al. 2019)
*Lactobacillus plantarum* CIDCA 83114, *Lactobacillus kefir* CIDCA 8348, *Lactococcus lactis* subsp *lactis* CIDCA 8221, *Kluyveromyces marxianus* CIDCA 8154, *Saccharomyces cerevisiae* CIDCA 8112	Milk kefir	Prevention of invasion of cell Hep-2 by *Shigella.* Protection of Vero cells from *Clostridium difficile* toxins.	(Bolla 2011)
*Lactobacillus plantarum* CIDCA 8327*, Lactobacillus kefir* CIDCA 8348, *Kluyveromyces marxianus* CIDCA 8154	Cheese fermented with kefir grains	In vitro inhibition of *Escherichia coli* and *Salmonella enterica* strains. Inhibitory effect and ability to protect enterocytes of adhesion and invasion of *Salmonella enteritidis.*	(Londero 2012)
*Lactobacillus brevis, Lactobacillus pentosus, Lactobacillus plantarum*	Traditional fermenting Moroccan green olives	Antifungal activity against *Penicillium* sp. Inhibition zones against *Candida pelliculosa.* Antibacterial effect against gram-positive and gram-negative bacteria.	(Abouloifa et al. 2020)
*Lactobacillus plantarum 81, Lactobacillus plantarum 90*	Fermentation process of “Cupuaçu” (*Theobroma grandiflorum*)	Production of diffusible inhibitory compounds and co-aggregation. Anti-inflammatory pattern of immunological response.	(Ornellas et al. 2017)
*Lactobacillus plantarum M-13*	*Kalarei* (indigenous cheese-like fermented milk product)	Antibacterial activity against *Campylobacter* sp. J1, *S. aureus* P07, *B. cereus* PS1, *Klebsiella* sp. KS19, *E. faecalis* FS03*, S. pneumoniae* P1.	(Gupta and Bajaj 2017)
*Lentilactobacillus hilgardii*, *Lentilactobacillus buchneri, Saccharomyces cerevisiae*	Water kefir	Antimicrobial activity against *P. larvae* and *A. apis.*	(Rodríguez et al. 2023)

It is apparent that aspects of food processing by honey bees is dependent on beneficial microbes. This co-adapted balance can, however, be upset by exposure to agrochemicals or other environmental toxins, leading to a scenario of “missing microbes” in honey bees characterized by impaired digestion and immunity (Maes et al. 2016, Daisley et al. 2020a). Kakumanu et al. (2016) showed that beneficial lactic acid bacteria (namely *Lactobacillus* spp. and related taxa in Lactobacillales) and yeasts (*Metschnikowia* spp. and related taxa in Saccharomycetes) were depleted after in-hive exposure to a common fungicide (chlorothalonil) as well as insecticide (coumaphos). Given the extent of pesticide exposure in agroecosystems (Traynor et al. 2021), it is likely that the homeostasis of plant-pollinator-microbe interactions has been negatively impacted at a systems level. Accordingly, the supplementation of microbes from fermented foods to offset these negative impacts and promote digestive function in honey bees is a promising area of research.

### Approaches to Support or Restore Healthy Pollen Fermentation

The screening of functional food items to improve bee health has so far been predominantly limited to investigations on bee bread. Di Cagno et al. (2019) developed a protocol to ferment bee-collected pollen and showed that a simple starter culture of *Apilactobacillus kunkeei* and *Hanseniaspora uvarum* could effectively emulate the spontaneous yeast-lactic fermentation of bee bread. Functionally, these starter strains increased digestibility and bioavailability of nutrients while minimizing the uncontrolled growth of contaminant microbes in the raw pollen samples. Others have also evaluated more complex starters (containing various fructophilic lactic acid bacteria) for purposes of controlled beebread fermentation and the practical maintenance of honey bee stocks in apiculture (Ispirli and Dertli 2021). Although both approaches are promising, large scale field studies are needed to confirm their benefit and ultimate feasibility.

Indirectly, several probiotic studies also support the idea that the fermentation of pollen or pollen-substitutes may produce desirable effects on colony level health. For example, Maruscakova et al. (2020) found that supplementing a pollen suspension containing *Lactobacillus brevis* could improve immune function and microbial balance in the hive. Similarly, a probiotic yeast study revealed that supplementing honey bees with pollen broth fermented by *Aureobasidium melanogenum* led to an upregulation of nutrition-related gene expression (Hsu et al. 2021). It is often difficult to interpret the exact mechanism of benefit in probiotic studies though, as the nutritional composition of the fermentation matrix is not typically analyzed. Daisley et al. (2023) did show however that 3 strains of lactic acid bacteria could improve protein digestibility of a pollen patty supplement (2–20% increase in 11 amino acids) and that these changes were ultimately associated with significant colony level growth-promoting effects. Thus, the evaluation of nutrient composition has potential to reveal mechanistic insight and should be considered in future honey bee probiotic and fermented food studies.

### Water Kefir—A Potential Source of Beneficial Microbes for Optimizing Nectar Processing

**Fig. 1.**
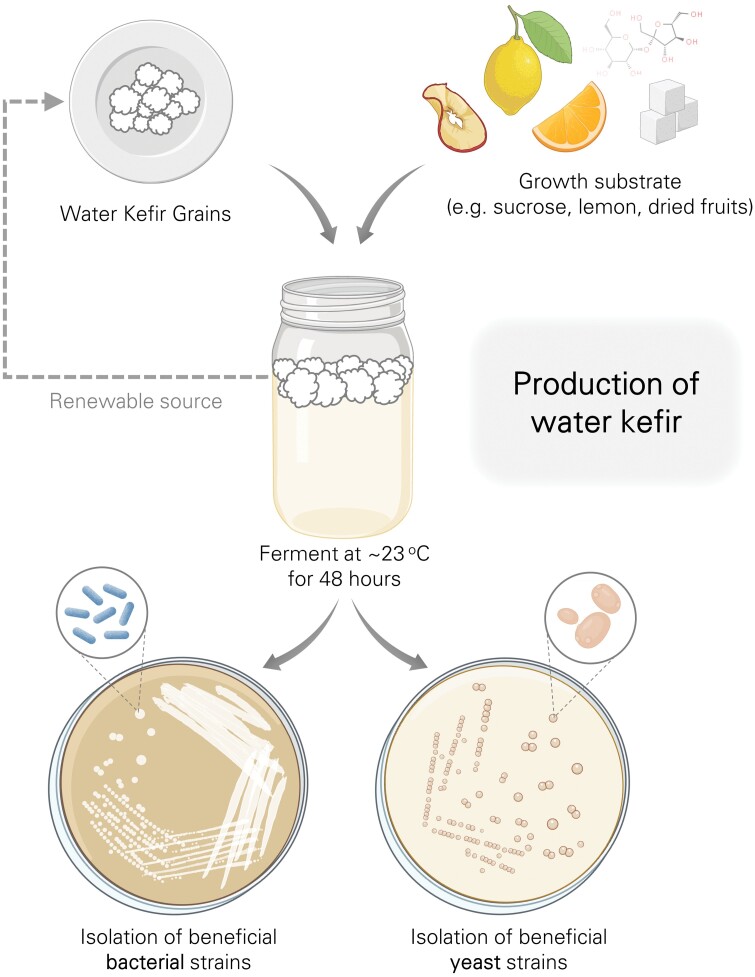
Production of water kefir. Kefir grains are added to a solution of water, sugar, dry fruits, and lemon and fermented at 23 °C ± 2 for 2 days. After filtering, a carbonated, acidic, slightly alcoholic beverage is obtained. Strains of lactic acid bacteria and yeasts can be isolated from water kefir.

Plant-derived nectar constitutes a large portion of the honey bee diet, but its consumption poses a major risk in terms of disease transmission to the hive. Compared to studies on pollen and beebread, there has been little investigation on optimizing nectar fermentation to reduce disease risk. One reason could be the inherent challenges of studying nectar microbiomes, which can change rapidly, as well as the inability to track or control all nectar sources accessible by honey bees in each environment. Nonetheless, sucrose syrup mixtures are a common supplement used in beekeeping that are meant to emulate nectar and prevent starvation of honey bees during lean periods. Although readily consumed by the bees, sucrose syrup lacks many of the chemical characteristics of nectar, is prone to spoilage, and has potential to stimulate growth of pathogens in the hive. Thus, this common form of supplemental feed is a prime candidate for fermentation via the inoculation of beneficial microbes. One potential source proposed as a suitable starter culture is water kefir (Fernández et al. 2017, Del Prado 2019, Rodríguez et al. 2019) – a fermented solution of water, sugar, and dried fruits (Fig. 1). During fermentation, the microbial community uniquely forms what are known as kefir “grains”, which are macroscopic structures formed primarily by lactic acid bacteria and yeasts. These grains resist contamination and can be used for inoculation of subsequent batches thereby supporting sustainable production of the water kefir.

Water kefir and plant nectar share some intriguing parallels in terms of their nutritional profile, microbiota composition, and relevance to honey bees (Table 2). In terms of nutrient content, water kefir and plant nectar are rich sources of vitamins (e.g., vitamin C), secondary metabolites (e.g., polyphenols, quercetin), and other micronutrients (Nicolson 2022, Constantin et al. 2023). Additionally, both are low in proteins and lipids but high in carbohydrates, particularly simple sugars that are easily digested by bees. Sucrose (table sugar) typically serves as the primary energy source for the microbial communities in water kefir, which is broken down into glucose and fructose – key monosaccharides that are vital for sustaining flight and overall metabolic activities of honey bees. A common practice in beekeeping is to catalyze this breakdown by performing nonenzymatic hydrolysis of sucrose syrups (i.e., inverted sugar syrup) via application of excessive heat and acidic conditions, but this process creates toxic compounds such as hydroxymethylfurfural that can be harmful to bee health (Frizzera et al. 2020). Thus, enzymatic conversion via microbial metabolism represents a viable alternative with a range of additional health benefits.

**Table 2.** Gross similarities between plant nectar and water kefir Microbial composition can vary greatly depending on the species of plant and choice of fermentation substrate. As such, these comparisons should serve only as a general guideline of the potential microorganisms that may be detected

	Plant nectar	Reference	Water kefir	Reference
Chemical properties				
pH	6.1–8.0	(Lenaerts et al. 2016)	3.76 ± 0.03 to 5.88 ± 0.05 3.66 ± 0.02 to 4.00 ± 0.03	(Laureys et al. 2017) (Gamba et al. 2021)
Sugar content	10–70% w/w	(Nicolson 2022)	221 ± 3 and 228 ± 2 g liter^−1^	(Laureys et al. 2017)
Microbial properties				
Lactic acid bacteria	*Lactococcus garvieae Apilactobacillus kunkeei*	(Martin et al. 2022)	*Lactobacillus paracasei* *Lb. nagelii* *Lb. satsumensis* *Leuconostoc mesenteroides*	(Laureys et al. 2017)
			*Lactobacillus hilgardii* *Lactobacillus casei*	(Lynch et al. 2021) (Moretti et al. 2022)
Other bacteria	*Acinetobacter* *Gluconobacter* *Gluconacetobacter Acetobacteraceae*	(Morris et al. 2020)	*Acetobacter orientalis* *Gluconobacter cerinus* *G. roseus/oxydans*	(Laureys et al. 2017)
Yeasts	*Metschnikowiaceae Saccharomycetaceae*	(Morris et al. 2020)	*Saccharomyces cerevisiae Zygotorulaspora florentina* *Dekkera anomala* *Candida boidinii* *Pichia membranifaciens,* *Wickerhamomyces anomalus* *Hanseniaspora*	(Lynch et al. 2021)

Regarding microbiome profiles, both plant nectar and water kefir harbor a dynamic consortia of lactic acid bacteria and yeasts that collaboratively ferment sugars and transform plant secondary compounds into a range of bioactive microbial metabolites. Though highly variable, total yeast loads in nectar and water kefirs are approximately 10^8^ and 10^7^ CFU/ml, where bacterial loads can reach 10^10^ and 10^8^ CFU/ml, respectively (Lynch et al. 2021, Martin et al. 2022). Both also exhibit low pH from the presence of organic acids and alcohols produced during fermentation. Nectars vary greatly in their pH but are generally acidic, with apricot (*Prunus armeniaca*) nectar, for example, possessing a pH of 3.4–3.6 (Reale et al. 2020). Whereas water kefir produced from dried apricots, or figs, has a pH of ~3.45–3.53 (Tireki 2022, Zannini et al. 2022). Notably, these estimates are in line with the pH of 3.56–3.66 for honey (Živkov Baloš et al. 2023).

Given these similarities and potential to improve honey bee digestion, water kefir holds promise to replace or augment standard sucrose syrups used in beekeeping. There is also potential to mine water kefir for the isolation of strains exhibiting probiotic properties of interest. Rodríguez et al. (2023) showed that several strains of lactic acid bacteria isolated from water kefir (including *Lentilactobacillus hilgardii* and *Lentilactobacillus buchneri*) could inhibit growth of the honey bee larval pathogen, *P. larvae* and *A. apis*, under in vitro conditions. However, further studies under field trial conditions are needed to determine whether these effects can have an impact on honey bee disease outcomes at the colony level.

### Potential Issues With Microbial Manipulations

Caution is warranted when isolating select strains exhibiting a specific beneficial trait, as opposed to multi-strain communities present in fermented bee food items, such as beebread and water kefir. Good et al. (2014) showed, for example, that honey bees avoided nectar experimentally inoculated with *Asaia astilbis*, *Erwinia tasmaniensis*, or *Lactobacillus kunkeei*, whereas foraging preference was unaffected by inoculation with the yeast, *Metschnikowia reukaufii*. Presumably, this avoidance behavior was to do with the rapid alterations of nectar chemistry caused by bacterial inoculation, as opposed to yeast which grow more slowly (Good et al. 2014). Pozo et al. (2021) demonstrated in bumble bees that the yeast *Wickerhamiella bombiphila* and the bacteria *Rosenbergiella nectarea* had a strong effect in terms of stimulating colony growth, whereas another yeast (*Torulaspora delbrueckii*) or a combination of yeast and bacteria showed less of a benefit on colonies. Other studies likewise provide good examples of bacterial manipulations that show no beneficial effects (Johnson et al. 2014, Stephan et al. 2019) or even negative impacts (Andrearczyk et al. 2014) on bee health. Further, feeding nonnative bacteria can trigger immune responses (Evans and Lopez 2004), which may or may not be beneficial. The role for probiotics to support bee gut microbial health is promising (Motta et al. 2022) but each study needs to be carefully interpreted, and overall future studies are needed to confirm a benefit in these approaches before applying them in commercial beekeeping practices (Damico et al. 2023).

## Concluding Remarks

Honey bees play a crucial role in crop pollination and sustaining our food supply but face challenges such as high overwinter mortality rates and the adverse effects of antibiotics used for disease control. Probiotics, especially lactic acid bacteria and select yeasts, are emerging as a promising alternative to antibiotics for management of bee diseases. Probiotics can enhance bee health through several mechanisms such as boosting immune responses and supporting the endogenous microbiome. Fermented products such as beebread and kefir also represent sources of potentially novel beneficial microbes to improve bee nutrition, resilience, and survival. Honey bees naturally rely on fermentation to process pollen and nectar, but exposure to agrochemicals can disrupt this balance through depleting the symbiotic microbial communities involved, leading to digestive and immunity issue. By incorporating fermented foods and water kefir into beekeeping practices, beekeepers may potentially mitigate these negative impacts and promote the overall health of honey bee colonies, although further field studies are urgently needed to validate the efficacy of these approaches.
